# The role of mechanical circulatory support as destination therapy for ambulatory heart failure

**DOI:** 10.21542/gcsp.2016.24

**Published:** 2016-09-30

**Authors:** James K. Kirklin

**Affiliations:** Department of Surgery, University of Alabama at Birmingham, Birmingham, AL, USA

## Abstract

Continuous flow technology has dramatically improved patient survival during ventricular assist device (VAD) therapy in recent years. Health-related quality of life is improved by at least two years. Despite remarkable progress in this field, major adverse events during VAD support limit the effectiveness of this therapy and present major barriers to its extension to ambulatory advanced heart failure patients. The pace of progress will depend on improvements in both the adverse event profile and development of semi-quantitative methodology to calculate and display a composite of survival and health-related quality of life.

## Introduction

Since the earliest iterations of mechanical circulatory support (MCS) devices, engineers and visionary surgeons have contemplated truly long-term support of the failing circulation that could be applied before the terminal stages of heart failure. However, even after the REMATCH trial demonstrated a significant survival advantage for desperately ill advanced heart failure patients compared to medical therapy,^1^ the adverse event profile was too unfavorable to justify application in less sick patients. A major paradigm shift emanated from the introduction of rotary pump technologies that gradually dominated the MCS landscape.

## Current outcomes with MCS therapy

**Figure 1.**
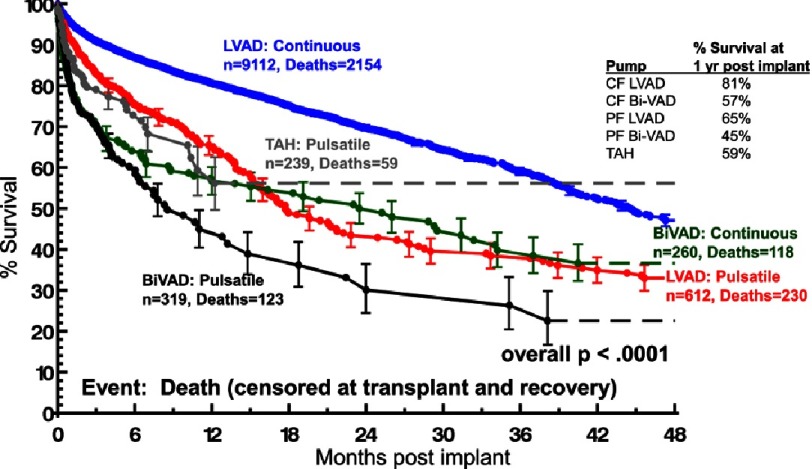
Kaplan-Meyer survival estimates for left ventricular assist devices (LVAD) and bi-ventricular assist devices (BIVAD) stratified by device types. CF, continuous flow; pf, pulsatile flow; TAH, total artificial heart. Error bars ± 1S.E. Patients are censored at transplant and recovery.^1^

**Figure 2.**
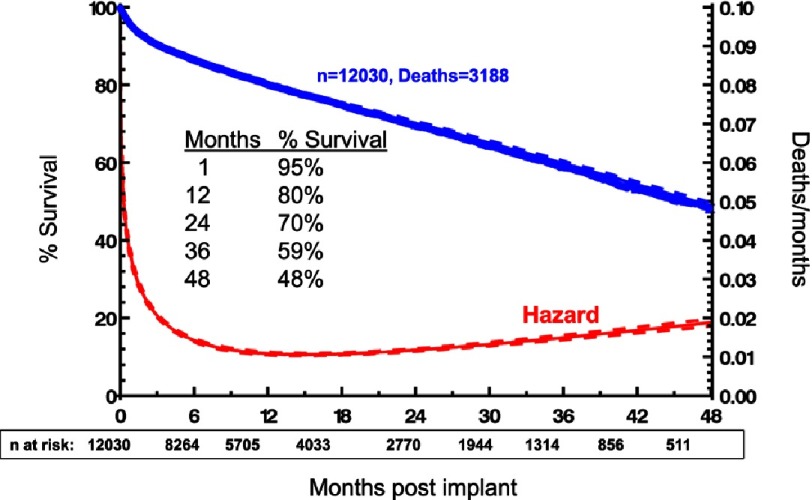
Parametric survival curve and associated hazard function with the 70% confidence limits for survival after implantation of a continuous flow left ventricular assist device (LVAD) or biventricular assist device (BIVAD). The number of patients at risk during each time interval is indicated below.^2^

The survival advantage of isolated left ventricular support with a durable continuous flow device compared to prior pulsatile technology has been well documented in the United States NHLBI-sponsored Interagency Registry for Mechanically Assisted Circulatory Support (INTERMACS). (Figure 1)^2^. With continuous flow pump technology, current 1- and 2-year expected survival rates are 80% and 70% respectively (Figure 2)^2^.

## Destination therapy

Although the application of mechanical circulatory support as long-term destination therapy (DT) has increased in the recent era (Table 1)^2^, the majority of DT patients have progressive cardiac deterioration and/or inotrope dependence at the time of implant. Less than 25% of implants are in patients with truly ambulatory heart failure (INTERMACS Levels 4–7)^3^ (Table 2).

**Table 1:** CF, continuous flow.

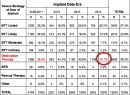

**Table 2:** 

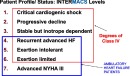

One of the most pressing groups of ambulatory advanced heart failure patients are those patients who have been accepted for cardiac transplantation, but whose status is not sufficiently urgent to justify the highest priority for donor allocation. Such patients often wait a year or more for a suitable donor.

**Table 3:** 

	Early hazard		Late hazard	
Risk Factors for Death	Hazard ratio	*p*-value	Hazard Ratio	*p*-value
Demographics			
Age (older)	1.03	<.0001	2.75	.008
Female	1.32	<.0001		
BMI (higher)	1.10	<.0001		
Blood Type Not O			1.24	.004
Clinical Status			
History of Stroke	1.33	.03		
Ventilator	1.25	.02		
ICD	1.30	.0001		
INTERMACS Level 1	1.55	<.0001		
INTERMACS Level 2	1.37	<.0001		
NYHA 4			1.23	.03
Destination Therapy	1.23	<.0001		
Non-Cardiac Systems			
Albumin (lower)	1.14	.0007		
Creatinine (higher)	1.06	.04	1.15	.002
Dialysis	2.34	<.0001		
BUN (higher)	1.05	<.0001		
Right Heart Dysfunction			
Right Atrial Pressure (higher)	1.13	.0004		
RVAD in same operation	2.57	<.0001		
Bilirubin (higher)	1.48	<.0001		
Surgical Complexities			
History of cardiac surgery	1.24	.003		
History of CABG	1.17	.04		
Concommitant Cardiac Surgery	1.26	<.0001		

**Notes.**

LVAD left ventricular assist device BiVAD biventricular assist device BMI body mass index BUN blood urea nitrogen RVAD right ventricular assist device ICD implantable cardioverter defibrillator CABG coronary artery bypass graft

**Figure 3.**
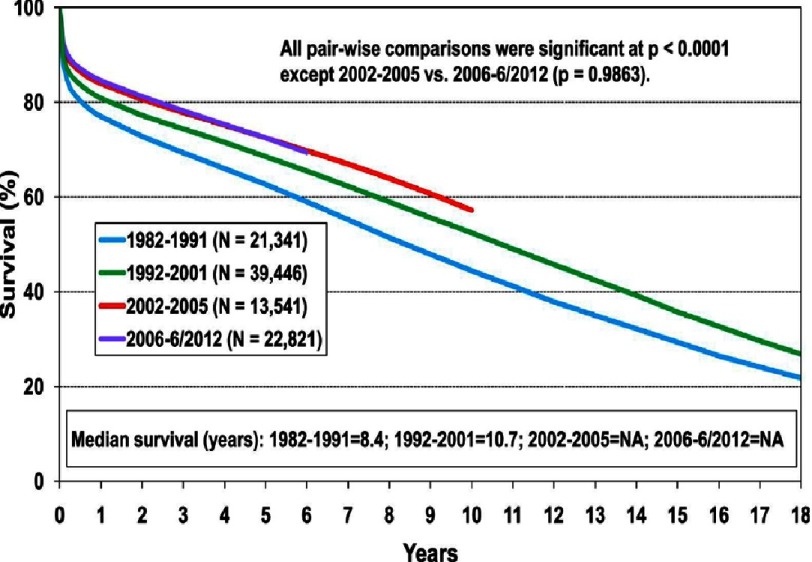
Survival curves for adult patients undergoing heart transplantation, stratified by era; from the Registry of the International Society for Heart and Lung Transplantation. Reproduced with permission.^4^

According to data from the International Society for Heart and Lung Transplantation (ISHLT), the average survival rate following cardiac transplantation is approximately 80% at 2 years (Figure 3)^4^. Therefore, for chronic mechanical circulatory support to be a viable alternative for potential transplant recipients, 2-year survival with mechanical circulatory support would need to at least approach 80%.

**Figure 4.**
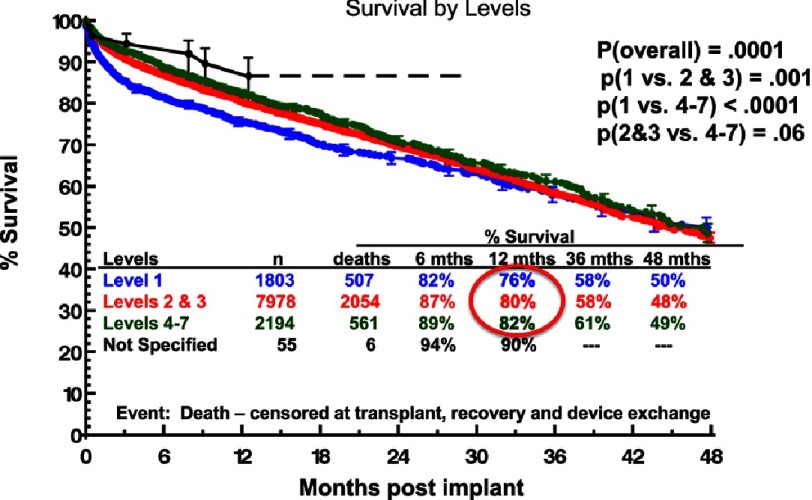
Kaplan-Meyer estimate for survival with continuous flow left ventricular assist device (LVAD) support with a strategy of destination therapy, stratified by INTERMACS Level at implant. Data obtained from the INTERMACS database.^2^

**Figure 5.**
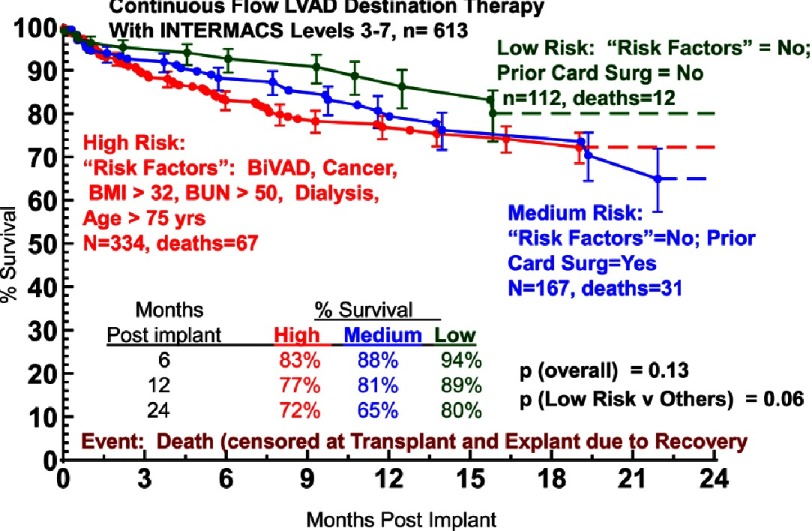
Kaplan-Meyer Survival estimate for patients receiving continuous flow left ventricular assist device (LVAD) therapy with a strategy of destination therapy, stratified by risk profile.^5^

A multi-variable analysis based on INTERMACS data for survival following implantation of durable continuous flow pumps indicates that patients with ambulatory heart failure have better expected survival than patients who are hemodynamically unstable (Table 3).^3^ This survival benefit is most apparent in the first 3 months following device implantation (Figure 4).^2^ In a study of INTERMACS patients undergoing destination therapy, a lower risk group of patients in INTERMACS Levels 3-7 was identified who did not have important right ventricular failure, important renal dysfunction, or a history of prior cancer; were under age 75 years and had no prior major cardiac operations. (Figure 5).^5^ This lower risk group represented nearly 20% of Level 3–7 INTERMACS patients who underwent destination therapy. The 1- and 2-year survival in this patient subset was 89% and 80%, respectively.

However, further analyses are needed since patients who currently receive DT are not comparable to standard heart transplant patients; DT patients currently must be considered ineligible for heart transplantation because of associated risk factors. Thus, the current population of DT patients has additional risk factors for mortality that may not be present in the transplant population. Any potential triage algorithm is further complicated by the reality that many risk factors (including renal dysfunction, right heart failure, previous cardiac surgery, and advanced age) that might identify a higher risk group for heart transplantation also identify a higher risk group for mechanical circulatory support (Tables 4 and 3)^6,2^. Therefore, it is not evident that simply triaging higher risk transplant patients to VAD therapy will drive an effective treatment algorithm.

**Table 4:** Risk factors for death (CTRD, 1990–2008)

• Date of transplant • Age • Pulmonary vascular resistance • Number of sternotomies • Etiology of cardiomyopathy • Pre-transplant serum creatinine • Creatinine clearance at listing • Mean RA pressure at pre-tx cath • Non-parous female • History of smoking • History of peripheral vascular disease	• VAD support at the time of transplant • Donor age • Donor heart ischemic time • Donor medical history of diabetes • Male donor • Donor medical history of hypertension • Recipient-Donor BMI difference • African American recipient • Pre-tx history of diabetes • Recipient percent ideal body weight

**Figure 6.**
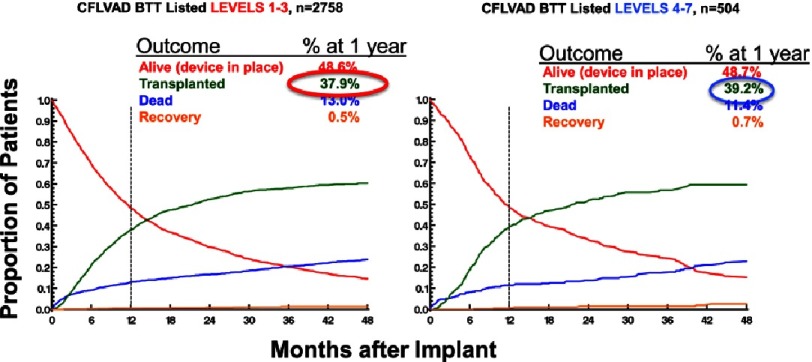
Competing outcomes depictions for patients receiving continuous flow LVAD (CFLVAD) bridge to transplant (BTT) therapy, separated by INTERMACS Level at implant. At any given time-point in the competing outcomes depiction, the proportion of each outcome event sums to a total of 1.0.^2^

If ambulatory heart failure patients could be routinely well supported with LVAD therapy, one might also consider a pathway whereby these less sick LVAD patients received improved access to heart transplantation compared to patients who were sicker at the time of LVAD implant. However, data from INTERMACS fails to suggest any such advantage. A competing outcomes analysis indicates that patients in Levels 4 through 7 have only a very small increase in the likelihood of heart transplantation within one year of implant compared to patients implanted in Levels 1 through 3 (Figure 6)^2^.

## Failure of the REVIVEIT trial

The REVIVEIT trial was a United States randomized clinical trial, supported by the National Heart, Lung, and Blood Institute (NHLBI), designed to examine the impact of mechanical circulatory support compared to optimal medical therapy for a group of patients with ambulatory advanced heart failure who were ineligible for cardiac transplantation. The HeartMate II device (Thoratec Inc.; Pleasanton, CA) was selected as the continuous flow pump, and the mechanical circulatory support community and the NHLBI felt that equipoise existed for designing a clinical trial with mechanical circulatory support devices in this less ill ambulatory advanced heart failure population.

**Figure 7.**
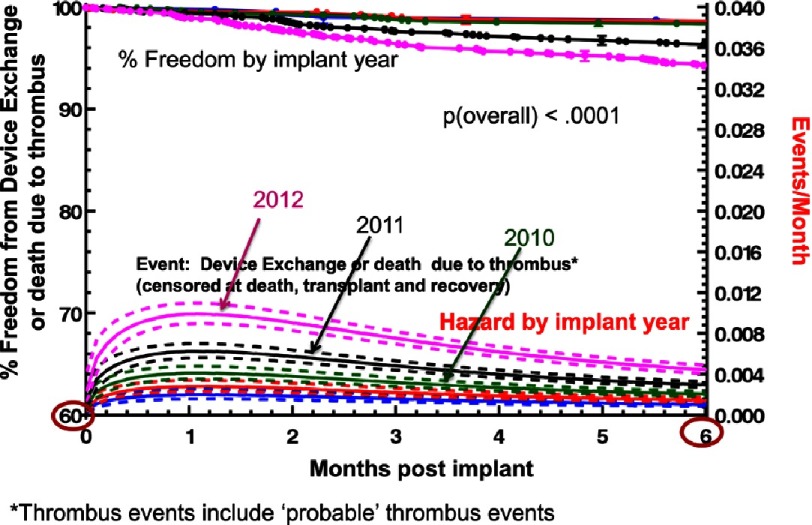
Kaplan-Meyer estimate and hazard function curves for freedom from device exchange or death due to pump thrombosis. The dashed lines indicate the 70% confidence limits.^7^

**Figure 8.**
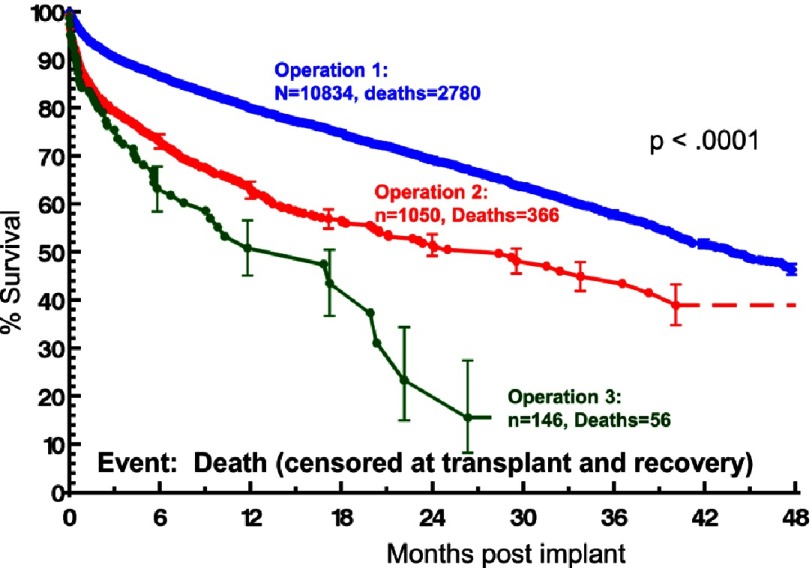
Kaplan-Meyer survival estimate for patients undergoing operations for implantation for continuous flow left ventricular assist devices (LVAD) in the HeartMate II pump thrombosis analysis. Operation 1 indicates the first implant. Operation 2 indicates the first pump exchange. Operation 3 represents the 2^nd^ pump exchange. BIVAD, biventricular assist device.^2^

However, despite a rigorous clinical trial design and multi-stage screening, in the end the trial was terminated because of the disruption of equipoise over concerns about adverse events and specifically pump thrombosis. Before the initial randomized patient could receive device therapy, an increasing rate of pump thrombosis was noted with this device. A detailed INTERMACS analysis identified a progressive increase in the hazard for pump thrombosis resulting in death or pump exchange from 2010 through 2012 (Figure 7)^7,8^. Notably, the mortality associated with pump exchange exceeded 25% at 6 months (Figure 8)^2^.

## Quality of life

**Figure 9.**
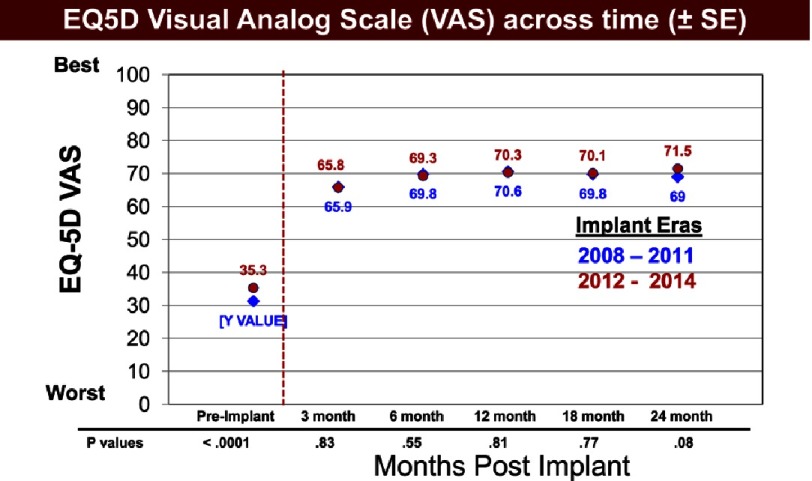
Visual analog scale (VAS) at specified time points before and after VAD implant among patients in the INTERMACS database.^2^

**Figure 10.**
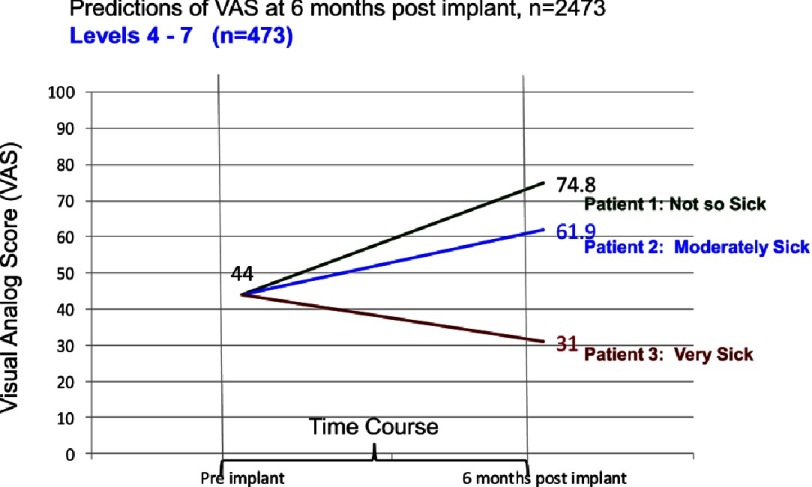
Change in visual analog score (VAS) from before to 6 months after VAD implant. The 3 lines depict patients with different post-implant adverse event burdens^9^ (see Table 5).

For many patients with advanced heart failure, their quality of life while supported by a durable pump is nearly or equally as important as the survival benefit. Grady and colleagues have firmly documented the improvement in quality of life with mechanical circulatory support, a benefit that is maintained for at least two years (Figure 9)^2,9^. Additional studies have documented the important impact of adverse clinical events on the relative improvement in quality of life following device implantation. In an INTERMACS study correlating adverse events during the first 6 months and quality of life, events such as renal dysfunction, respiratory failure, neurologic events, and infection negatively impacted quality of life 6 months following implantation (Table 5)^9^ (Figure 10).

**Table 5:** Factors associated with a change in VAS (6 months – Pre-implant), n = 2748. Negative coefficients indicate the decrement in change. The Intercept indicates the amount of change (improvement) for a patient with no ‘risk factors’.

Risk factors	Estimates (SE)	p value
Pre-implant conditions		
Level 1	5.0 (1.6)	0.002
BTT: Listed	−3.7 (1.2)	0.002
Pre COPD	−5.2 (1.8)	0.004
Ascites	−18.2 (5.0)	0.0003
Alcohol abuse	−3.9 (1.7)	0.02
Pre-implant VAS Score	−0.76 (0.02)	<0.0001
Clinical Course		
BTT: Unlikely at 6m	−9.7 (2.9)	0.0008
BTT: Mod likely at 6m	−4.9 (1.9)	0.01
NYHA4 at 6m	−14.2 (2.9)	<0.0001
Events within first 6 months		
Renal Dysfunction	−4.8 (2.5)	0.06
Respiratory Failure	−4.8 (1.8)	0.008
Neurological Dys	−5.5 (1.9)	0.004
Infection	−2.7 (1.1)	0.02

**Notes.**

Intercept = 64.3, R^2^ = 42%

## Adverse events in ambulatory heart failure patients who receive MCS therapy

**Figure 11.**
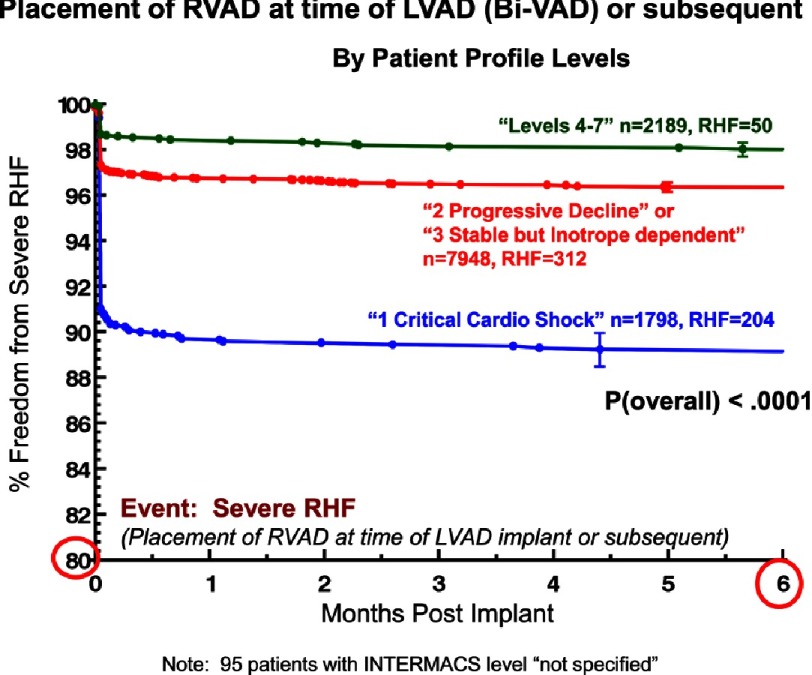
Kaplan-Meyer estimate for freedom from right heart failure (RHF) stratified by INTERMACS LEVEL at implant. Note that the vertical scale ranges from 80% to 100%. RVAD, right ventricular assist device; LVAD, left ventricular assist device; BIVAD, biventricular assist device.^2^

**Figure 12.**
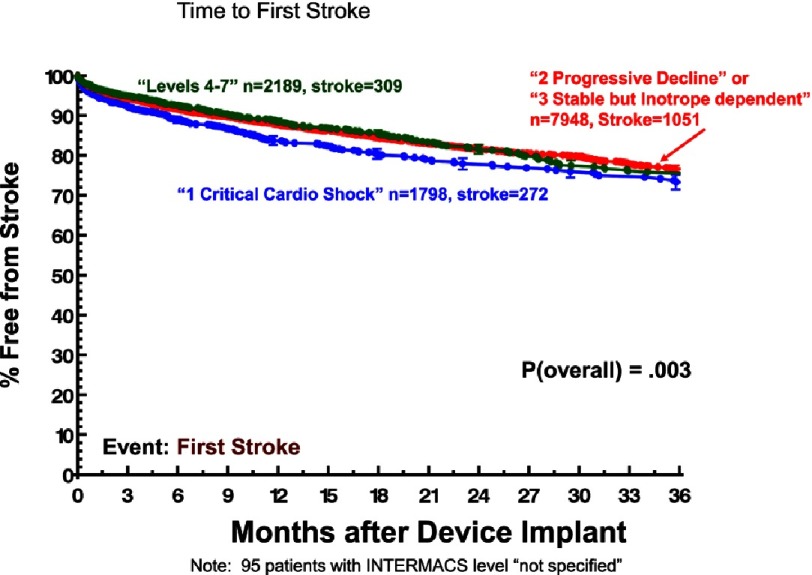
Kaplan-Meyer estimate for freedom from first stroke, stratified by INTERMACS Level at implant. CF, continuous flow; LVAD, left ventricular assist device; BIVAD, biventricular assist device.^2^

**Figure 13.**
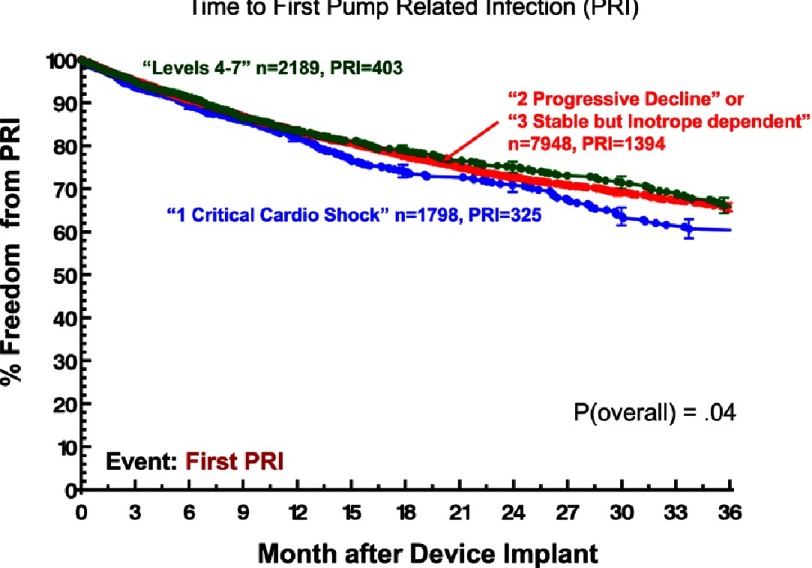
Kaplan-Meyer estimates for freedom from pump related infection (PRI), stratified by INTERMACS Level at implant.^2^

**Figure 14.**
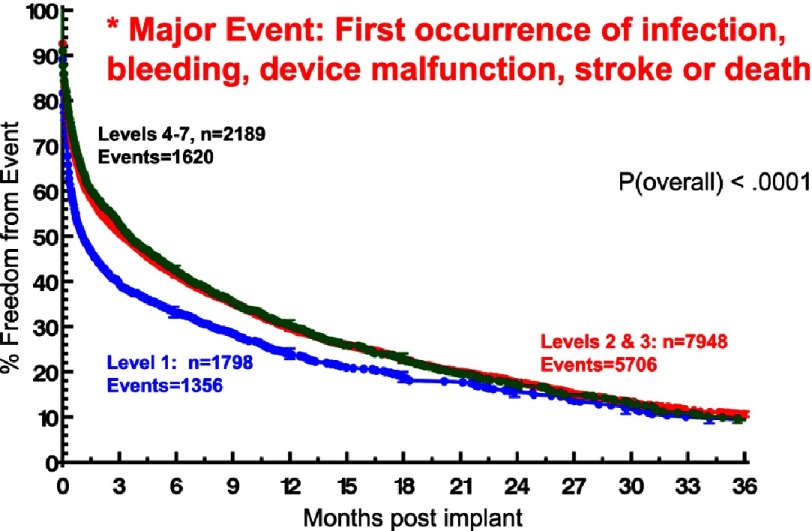
Kaplan-Meyer estimate for freedom from the combined major event of infection, bleeding, device malfunction, stroke, or death; stratified by INTERMACS Level at implant.^2^

**Figure 15.**
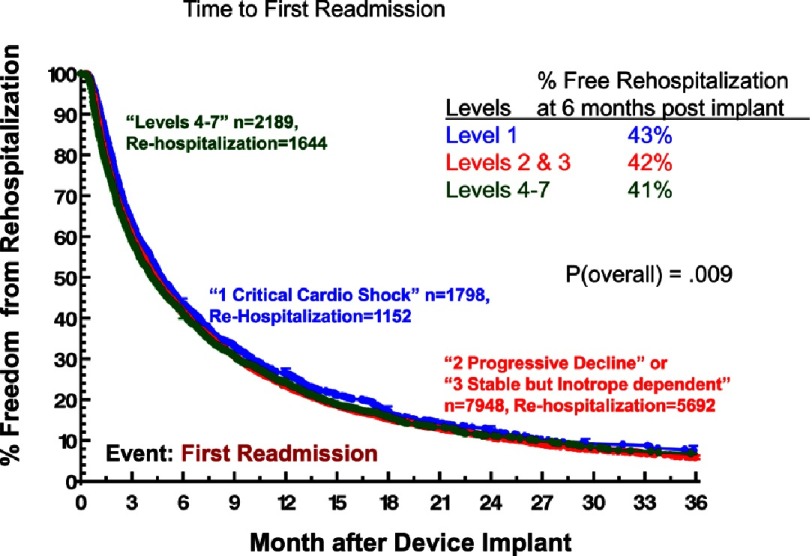
Kaplan-Meyer estimate for freedom from re-hospitalization, stratified by INTERMACS Level at implant.^2^

Although ambulatory heart failure patients at implant are less likely than more seriously ill patients to suffer severe right heart failure (Figure 11)^2^, they carry essentially the same risk for non-cardiac adverse events. Specifically, little or no difference between ambulatory and more seriously ill heart failure patients at VAD implant has been noted with respect to freedom from neurological events (Figure 12)^2^, pump-related infection (Figure 13)^2^, a combined major event end point (Figure 14)^2^, or the likelihood of hospital readmission (Figure 15)^2^.

## Quantifying the decision-making process

Time-related survival statistics are highly developed and readily quantifiable. However, such depictions, whether parametric or non-parametric, do not provide sufficient information for the patient with advanced heart failure to arrive at a truly informed decision about therapeutic choices, since quality of life and functionality are very important for most patients.

Currently, the complex domains of quality of life have not been sufficiently molded into quantative expressions to allow meaningful generation of suitable equations which could quantitatively describe a cumulative benefit of both survival and quality of life. When such methodology is available, semi-quantitative analyses may address such questions as “is your life better with a device?” or “are you functional with a good quality of life?”. Appropriate depictions of such questions could be combined with survival estimates to generate a composite depiction of survival/life quality benefit of one therapy compared to another.

**Figure 16.**
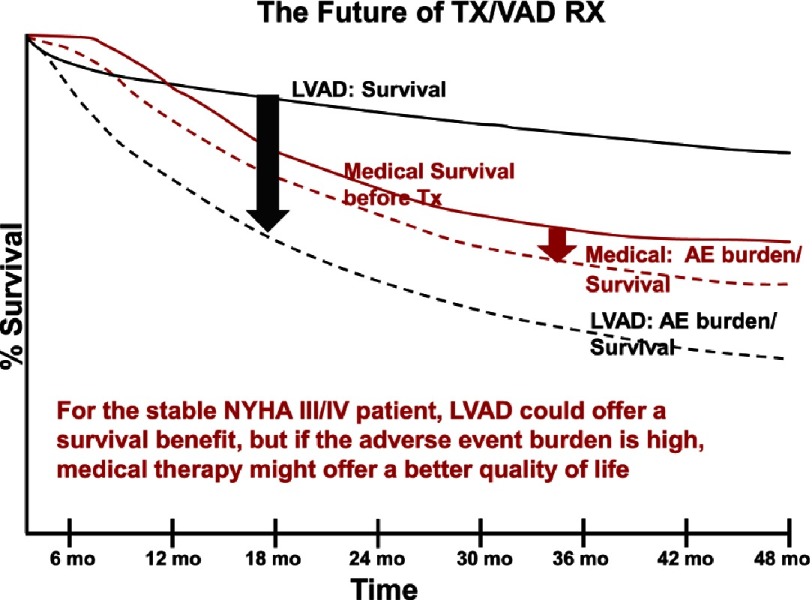
Hypothetical depiction of semi-parametric curves combining survival and adverse event burden following therapy with either a left ventricular assist device (LVAD) or medical therapy. The arrows indicate the decrement in combined survival and freedom from adverse event burden with each therapy. TX/VAD RX, transplant/ventricular assist device therapy; AE, adverse event.

The impact of quality of life, adverse events, and survival on the composite decision-making algorithm are illustrated in the following fictitious depictions. In the first instance (Figure 16) a stable ambulatory advanced heart failure patient is offered LVAD therapy vs. continued optimal medical treatment. With his/her risk profile, the expected time-related survival curves are shown. In this instance, however, the considerable additional adverse event burden with device therapy is such that the combined adverse event burden/survival curve is inferior to that of medical therapy.

**Figure 17.**
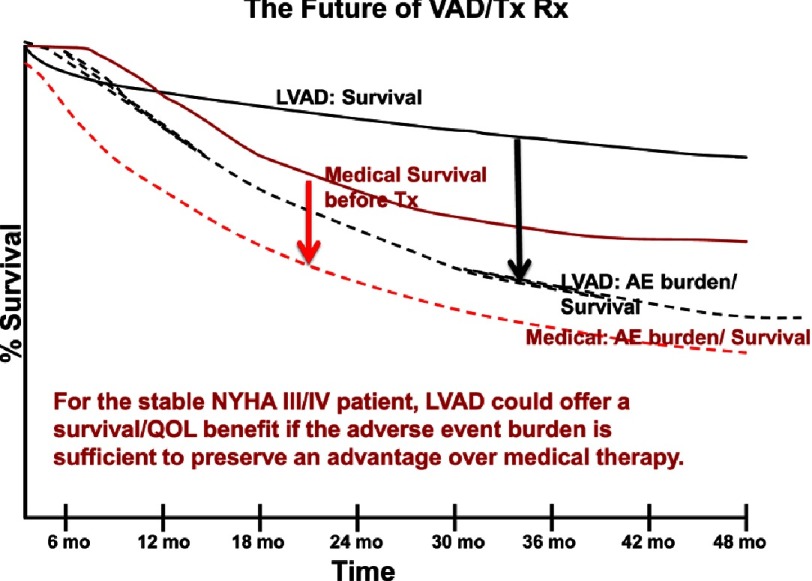
Same depiction as in Figure 16, with differing adverse event burdens.

However, with the same patient profile and expected survival curves, a reduced expected adverse event burdened predicts a superior overall adverse event burden/ survival curve compared to medical therapy (Figure 17). These hypothetical scenarios underscore the critical importance of reducing major adverse events if durable mechanical circulatory support is to become main stream therapy for advanced ambulatory heart failure.

## Conclusions

• In the current state of MCS technology, with the isolated exception of right heart failure, better “risk” status at implant provides limited or no “protection” against serious adverse events.
• Patients and providers will demand increasing quantification of expected “life satisfaction”, which will complement the calculation of survival after each therapy.
• The adverse event profile of individual MCS devices will dramatically affect the calculus of both the “life satisfaction survival score” and the calculation of “cost effective care”.
• Final recommendations will weigh heavily on time-related depictions (curves) of mathematical solutions to these outcome equations.
• It will be our charge to develop the language and find the equations to facilitate these complex decisions.

## References

[1] Rose EA, Gelijns AC, Moskowitz AJ, Heitjan DF, Stevenson LW, Dembitsky W, Long JW, Ascheim DB, Tierney AR, Levitan RG, Watson JT, Meier P, for the REMATCH Study Group (2001). Long-term Use of A Left Ventricular Assist Device for End-Stage Heart Failure. N Engl Med.

[2] Kirklin JK, Naftel DC, Pagani FD, Kormos RL, Stevenson LW, Blume ED, Myers SL, Miller MA, Baldwin JT, Young JB (2015). Seventh INTERMACS annual report: 15,000 patients and counting. J Heart and Lung Transplant.

[3] Kirklin JK, Naftel DC, Kormos RL, Stevenson LW, Pagani FD, Miller MA, Ulisney KL, Baldwin JT, Young JB (2011). Third INTERMACS Annual Report: The evolution of destination therapy in the United States. J Heart Lung Transplant.

[4] Lund LH, Edwards LB, Kucheryavaya AY, Benden C, Dipchand AI, Goldfarb S, Levvey BJ, Meiser B, Rossano JW, Yusen RD, Stehlik J, International Society for Heart and Lung Transplantation (2015). The Registry of the International Society for Heart and Lung Transplantation: Thirty-second Official Adult Heart Transplantation Report – 2015; Focus Theme: Early Graft Failure. J Heart Lung Transplant.

[5] Kirklin JK, Naftel DC, Pagani FD, Kormos RL, Stevenson L, Miller M, Young JB (2012). Long-term mechanical circulatory support (destination therapy): On track to compete with heart transplantation?. J Thorac Cardiovasc Surg.

[6] Tallaj JA, Pamboukian SV, George JF, Kirklin JK, Brown RN, McGiffin DC, Acharya D, Loyaga-Rendon R, Melby SJ, Bourge RC, Naftel DC (2014). Have risk factors for mortality after heart transplantation changed over time? Insights from 19 years of Cardiac Transplant Research Database study?. J Heart Lung Transplant.

[7] Kirklin JK, Naftel DC, Kormos RL, Pagani FD, Myers SL, Stevenson LW, Acker MA, Goldstein DL, Silvestry SC, Milano CA, Baldwin TJ, Pinney S, Eduardo RJ, Miller MA (2014). Interagency Registry for Mechanically Assisted Circulatory Support (INTERMACS) analysis of pump thrombosis in the HeartMate II left ventricular assist device. J Heart Lung Transplant.

[8] Kirklin JK, Naftel DC, Pagani FD, Kormos RL, Myers SL, Acker MA, Rogers J, Slaughter MS, Stevenson LW (2015). Pump thrombosis in the Thoratec HeartMate II device: An update analysis of the INTERMACS Registry. J Heart Lung Transp.

[9] Grady KL, Naftel DC, Myers SL, Kirklin JK (2015). Pre and Post-Operative Factors Relate to Change in Health-Related Quality of Life From Before to 6 Months Following LVAD Implantation: An INTERMACS analysis. Heart Lung Transplant.

